# Survival after a two-stage surgical approach in hepatopulmonary fusion: A case report

**DOI:** 10.1016/j.ijscr.2023.108467

**Published:** 2023-07-05

**Authors:** Maudy Aguilar Franco, Sergio Alzate-Ricaurte, Edgar Dario Alzate Gallego, Daniel Felipe Kafury, Ana Lucia Guzman Botero, Daniela Castaño Avila

**Affiliations:** a Fundación Valle del Lili, Department of Pediatric Surgery, Cra 98 Num. 18-49, Cali 760032, Colombia; b Fundación Valle del Lili, Centro de Investigaciones Clinicas, Cra 98 Num. 18-49, Cali 760032, Colombia; c Universidad ICESI, School of medicine, Cali, Colombia

**Keywords:** Congenital diaphragmatic hernia, Bochdalek, Hepatopulmonary fusion, Liver, Lungs

## Abstract

**Introduction:**

Congenital diaphragmatic hernias are a rare developmental defect. Pulmonary complications are more frequently seen in right sided defects (Partridge et al., 2016). Hepatopulmonary fusion is a rare and highly mortal malformation exclusively seen in right sided congenital diaphragmatic hernias marked by the fibrovascular fusion of the liver and lung.

**Presentation of case:**

A newborn male presented with respiratory distress and a 1-minute APGAR score of 7. A chest radiograph showed complete opacification of the right hemithorax, and a CT-Scan confirmed a congenital diaphragmatic hernia, an intrathoracic location of the liver and secondary pulmonary hypoplasia. 48 h after, intraoperative findings showed fusion of diaphragm, lung and liver tissue. Four months after, complete tissue division of the lower lobe from the fused liver segments VII/VIII and correction of the hernia defect was achieved. The patient was discharged from the hospital six months after.

**Discussion:**

Partial division of tissues is described as the safest and most successful approach to hepatopulmonary fusion. The tally of all cases reported worldwide until 2020 shows higher survival rates with complete division of tissues (Ferguson DM; Congenital Diaphragmatic Hernia Study Group, 2020) Reported cases lean towards one-session surgical interventions. A two-stage surgical approach allows an initial low surgical trauma to manage compressive effects on intrathoracic structures by herniary contents and a second time for tissue division in a non-critical patient, in this case leading to long-term survival.

**Conclusion:**

Hepatopulmonary fusion is a rare and highly lethal malformation with scarce information available. Future multicenter studies should compare different therapeutic options and search for outcomes including but not limited to mortality.

## Highlights

• Hepatopulmonary fusion is a rare and highly fatal malformation found in right-sided congenital diaphragmatic hernias.
• The data from all reported cases worldwide until 2020 indicates higher survival rates when complete division of tissues is performed.
• A two-stage approach allowed for an initial low-surgical-trauma to address intrathoracic compression, and tissue division in a non-critical patient.
• Due to a lack of comparative data on therapeutic approaches, conclusive evidence regarding the optimal management of hepatopulmonary fusion is unavailable.

## 1 Introduction

Congenital diaphragmatic hernias are a rare malformation with an incidence of 2.4–4.1 per every 10,000 births worldwide. It is characterized by the appearance of a diaphragmatic defect during development that allows herniation of abdominal organs to the thoracic cavity [3]. In terms of laterality, 80 % of cases are left sided, 15 % are right sided, and 5 % are present bilaterally [4]. Regarding location, most defects are posterior and lateral, also called Bochdalek hernias [3]. The clinical spectrum of Bochdalek congenital diaphragmatic hernias ranges from respiratory distress to severe pulmonary hypertension with fatal outcomes [4].

Hepatopulmonary fusion is a rare and highly mortal malformation exclusively seen in right sided defects, characterized by the fibrovascular fusion of the liver and lung, affecting 3 in every 1000 patients [5]. Although its pathophysiology has not been fully understood, two theories are accepted: the appearance of a diaphragmatic defect during organ development and a preceding fusion causing a diaphragmatic hernia [5]. A case report of a patient intraoperatively diagnosed with hepatopulmonary fusion in a right congenital diaphragmatic hernia managed with a two-stage surgical approach in an academic hospital is presented; 50 cases had been reported worldwide as of 2020, most of them with fatal outcomes [3]. This case report has been reported in line with SCARE criteria [6].

## 2 Presentation of case

A male infant was born at 37 weeks by Ballard's scale. Relevant to its medical history, obstetric prenatal ultrasounds and a fetal echocardiogram had previously shown a moderate tricuspid insufficiency with secondary dilation of the right atrium.

At birth via vaginal delivery, the patient had poor neonatal adaptation with a 1-minute APGAR score of 7, poor respiratory effort and cyanosis. Initially, free-flow oxygen was administered, followed by non-invasive ventilation without showing the desired clinical response; orotracheal intubation was indicated due to the persistence of respiratory distress. The patient was transferred to the Neonatal Intensive Care Unit. An echocardiogram showed mild valvular insufficiency of all cardiac valves, patent ductus arteriosus with bidirectional shunt, but an adequate ventricular function. A chest radiograph showed complete opacification of the right hemithorax, highly suggestive of a right congenital diaphragmatic hernia (Image A). A chest CT confirmed a right Bochdalek congenital diaphragmatic hernia and an intrathoracic location of the liver with secondary pulmonary hypoplasia (Image B). During the first 48 h after birth the patient developed obstructive shock requiring vasoactive and inotropic support.

**Image A:**
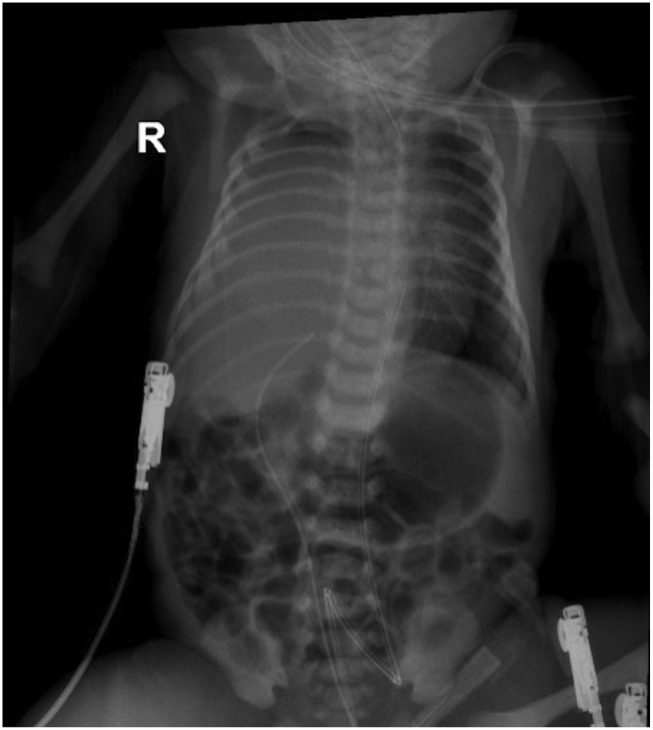
A chest radiograph was performed on day 1 after birth, which revealed complete opacification of the right hemithorax.

**Image B:**
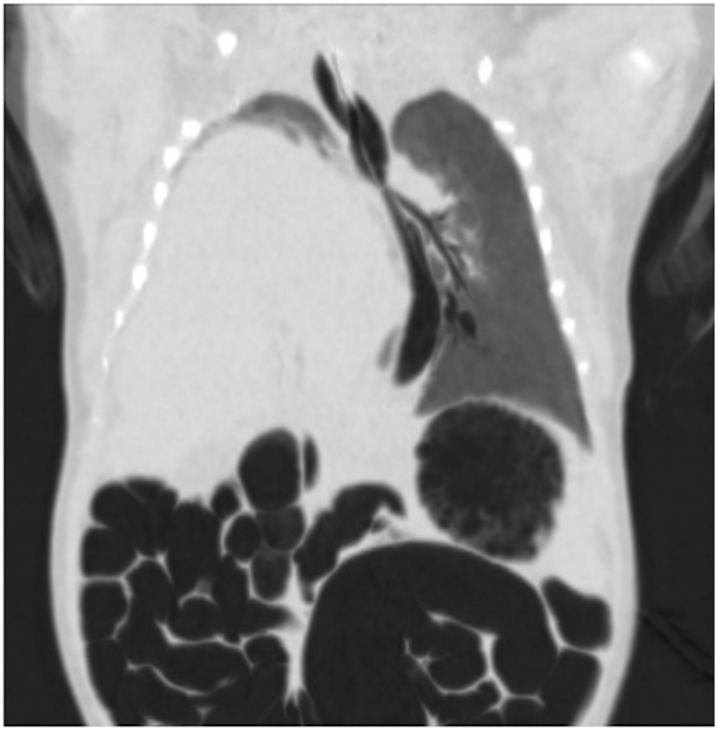
Confirmatory chest CT scan showing a right congenital diaphragmatic hernia with an intrathoracic location of the liver.

Two days after birth, an initial surgical session was performed; hernia correction was intended. An extended posterolateral thoracotomy at the seventh intercostal space was elected to facilitate exposure of the hernia sac and contents. Intraoperatively, a type D congenital diaphragmatic hernia (The Congenital Diaphragmatic Hernia Study Group) was found, with only minimal muscular borders identified in the posterolateral aspect. A thick hernia sac containing the incarcerated right hepatic lobe was seen, and no additional contents were detected. Notably, in its posterior aspect, a fusion of diaphragmatic tissue, the right lower lobe and segments VII/VIII of the liver was found. In its medial aspect, liver segments IVa and IVb showed adherence to the mediastinum in vicinity with the great vessels. A safe surgical plane for tissue division couldn't be achieved; partial reduction of the liver into the abdominal cavity and plication of the hernia sac with interrupted sutures of 2–0 nonabsorbable monofilament poly suture (ethylene terephthalate) was performed to manage its compressive effect on intrathoracic structures.

Three days after the first surgical session, an echocardiogram showed severe pulmonary hypertension and hypoplasia of the right pulmonary artery with no additional vascular abnormalities. The cardiovascular surgery department was called upon and pharmacological treatment to close the previously identified patent ductus arteriosus was implemented successfully. Nevertheless, the patient had a lengthy stay in the NICU, requiring prolonged nutritional, ventilatory, inotropic and vasoactive support. A second surgical session had to be postponed for four months. After clinical stability was achieved, the patient underwent a second surgical session via a right subcostal abdominal incision. Complete tissue division of the lower lobe from the fused liver segments VII/VIII and correction of the hernia defect using a monofilament polyester textile mesh covered with an absorbable hydrophilic film on one of its sides was carried out; a patch wasn't used. Division of adhered segments IVa and IVb to the mediastinum was not possible due to the inherent risk of injury to the great vessels. The patient had no postoperative complications. The patient was discharged from the hospital two months after the second surgical session and six months after birth.

One year after discharge, a ventilation-perfusion (VQ) scan showed an 8 % contribution of the right lung to pulmonary function, with only a functioning right superior lobe apex, however, no segmentary perfusion defects. The patient has attended follow-ups for the past three years and does not require oxygen therapy for daily life (Image C). No additional pulmonary function tests have been performed so far.

**Image C:**
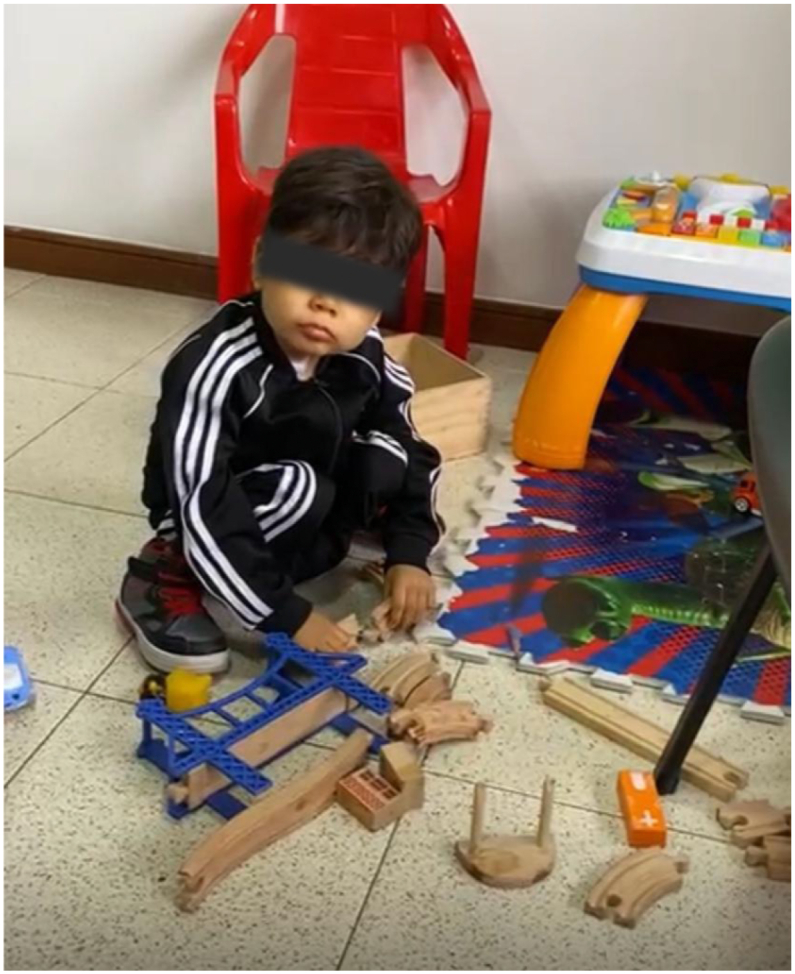
Patient at the three-year-old follow up.

## 3 Discussion

Congenital diaphragmatic hernias occur during development of the diaphragm, leading to an intrathoracic location of abdominal organs. Herniation of abdominal organs during pulmonary development leads to secondary pulmonary hypoplasia, compression of thoracic vascular structures and muscle hypoplasia of the pulmonary artery leading to severe pulmonary hypertension [7]. Hepatopulmonary fusion is a rare malformation, seen in this patient as a fibrovascular fusion between the herniated liver, the pulmonary parenchyma, and the diaphragmatic tissue. Data on mortality is unavailable due to its low incidence. However, most cases have fatal outcomes [8]. Although laterality in congenital diaphragmatic hernias has no effect on mortality, a greater incidence of pulmonary complications is seen in right sided hernias [1].

Surgical interventions in congenital diaphragmatic hernias are dictated by the severity of pulmonary injury and aim to reduce hernia contents and the primary closure of the herniary defect [9,10]. Cases of hepatopulmonary fusion provide an additional challenge due to the incapacity of attaining a safe surgical plane for tissue division and the high morbimortality seen with this malformation. Although Ferguson et al. propose a partial division of tissues as the safest approach while also associating it with better outcomes in their review of the CDHR database, the worldwide tally of cases up until 2020 shows greater survival rates with complete separation of tissues (45.4 % partial, 53.3 % complete) [2]. Performing complete liver, diaphragmatic and lung tissue separation and the early identification of the inherent risk associated with mediastinal adherence separation led to a safe and physiological repair. Nevertheless, a lack of data comparing both therapeutic approaches doesn't allow definite conclusions on the best approach to hepatopulmonary fusion.

Surgical approaches on reported cases lean towards hernia correction and division of tissues in a single surgical session [2,4]. A two-stage surgical approach, preconditioned on patient survival after the first surgery, allows for an initial low surgical trauma to manage compressive effects to intrathoracic structures by herniary contents and a second session with high surgical trauma for tissue division in a patient with an improved clinical status, in this case leading to long-term survival as the outcome. Future multicenter studies in reference centers should compare different therapeutic options and search for outcomes including but not limited to mortality. Emphasis on encouraging reporting worldwide should be made to enhance the pool of available information [11], which can then be analyzed and utilized to enhance patient care.

## Consent

Written informed consent was obtained from the patient’s guardian for publication of this case report and accompanying images. A copy of the written consent is available for review by the Editor-in-Chief of this journal on request.

## Ethical approval

The final version of this manuscript was submitted to the Fundación Valle del Lili ethics committee and approved for submission via Act Num. 04, 2023, Report case No. 664, IRB Approval No. 140-2023 on February 22, 2023.

## Funding

This research received no specific grant from any funding agency in the public, commercial, or not-for-profit sectors.

## Guarantor

Sergio Alzate-Ricaurte.

## Research registration number

None.

## CRediT authorship contribution statement

Maudy Aguilar Franco: Lead surgeon on the case. Design. Proof reading.

Sergio Alzate-Ricaurte: Writing the paper.

Edgar Dario Alzate Gallego: Accompanying surgeon on the case. Proof reading.

Daniel Felipe Kafury: Support in case description writing.

Ana Lucia Guzman Botero: Support in abstract writing.

Daniela Castaño Avila: Data collection.

## Conflict of interest statement

None.
